# Associations of Race/Ethnicity and Food Insecurity With COVID-19 Infection Rates Across US Counties

**DOI:** 10.1001/jamanetworkopen.2021.12852

**Published:** 2021-06-08

**Authors:** Mumbi E. Kimani, Mare Sarr, Yendelela Cuffee, Chang Liu, Nicole S. Webster

**Affiliations:** 1 School of Economics and Finance, University of the Witwatersrand, Johannesburg, South Africa; 2 School of International Affairs, Pennsylvania State University, University Park; 3 School of Economics, University of Cape Town, Rondebosch, South Africa; 4 College of Health Sciences, University of Delaware, Newark; 5 College of Education, Pennsylvania State University, University Park; 6 College of Agricultural Sciences, Pennsylvania State University, University Park

## Abstract

**Importance:**

Food insecurity is prevalent among racial/ethnic minority populations in the US. To date, few studies have examined the association between pre–COVID-19 experiences of food insecurity and COVID-19 infection rates through a race/ethnicity lens.

**Objective:**

To examine the associations of race/ethnicity and past experiences of food insecurity with COVID-19 infection rates and the interactions of race/ethnicity and food insecurity, while controlling for demographic, socioeconomic, risk exposure, and geographic confounders.

**Design, Setting, and Participants:**

This cross-sectional study examined the associations of race/ethnicity and food insecurity with cumulative COVID-19 infection rates in 3133 US counties, as of July 21 and December 14, 2020. Data were analyzed from November 2020 through March 2021.

**Exposures:**

Racial/ethnic minority groups who experienced food insecurity.

**Main Outcomes and Measures:**

The dependent variable was COVID-19 infections per 1000 residents. The independent variables of interest were race/ethnicity, food insecurity, and their interactions.

**Results:**

Among 3133 US counties, the mean (SD) racial/ethnic composition was 9.0% (14.3%) Black residents, 9.6% (13.8%) Hispanic residents, 2.3% (7.3%) American Indian or Alaska Native residents, 1.7% (3.2%) Asian American or Pacific Islander residents, and 76.1% (20.1%) White residents. The mean (SD) proportion of women was 49.9% (2.3%), and the mean (SD) proportion of individuals aged 65 years or older was 19.3% (4.7%). In these counties, large Black and Hispanic populations were associated with increased COVID-19 infection rates in July 2020. An increase of 1 SD in the percentage of Black and Hispanic residents in a county was associated with an increase in infection rates per 1000 residents of 2.99 (95% CI, 2.04 to 3.94; *P* < .001) and 2.91 (95% CI, 0.39 to 5.43; *P* = .02), respectively. By December, a large Black population was no longer associated with increased COVID-19 infection rates. However, a 1-SD increase in the percentage of Black residents in counties with high prevalence of food insecurity was associated with an increase in infections per 1000 residents of 0.90 (95% CI, 0.33 to 1.47; *P* = .003). Similarly, a 1-SD increase in the percentage of American Indian or Alaska Native residents in counties with high levels of food insecurity was associated with an increase in COVID-19 infections per 1000 residents of 0.57 (95% CI, 0.06 to 1.08; *P* = .03). By contrast, a 1-SD increase in Hispanic populations in a county remained independently associated with a 5.64 (95% CI, 3.54 to 7.75; *P* < .001) increase in infection rates per 1000 residents in December 2020 vs 2.91 in July 2020. Furthermore, while a 1-SD increase in the proportion of Asian American or Pacific Islander residents was associated with a decrease in infection rates per 1000 residents of −1.39 (95% CI, −2.29 to 0.49; *P* = .003), the interaction with food insecurity revealed a similar association (interaction coefficient, −1.48; 95% CI, −2.26 to −0.70; *P* < .001).

**Conclusions and Relevance:**

This study sheds light on the association of race/ethnicity and past experiences of food insecurity with COVID-19 infection rates in the United States. These findings suggest that the channels through which various racial/ethnic minority population concentrations were associated with COVID-19 infection rates were markedly different during the pandemic.

## Key Points

**Question:**

Are racial/ethnic population composition and food insecurity associated with COVID-19 infection rates?

**Findings:**

This cross-sectional study of 3133 US counties found that there was an association between race/ethnicity and COVID-19 infection rate, with an interaction with food insecurity in counties with large Black and American Indian or Alaska Native populations but not in counties with large Hispanic populations.

**Meaning:**

These findings suggest that public policy aimed at fighting COVID-19 should consider county-level food insecurity to better understand the social dynamics of the disease.

## Teaser

This cross-sectional study investigates the associations of race/ethnicity and food insecurity with COVID-19 infection rates in US counties.

## Introduction

The COVID-19 pandemic has taken a toll on health care systems globally. By mid-December 2020, more than 300 000 deaths and 18 million infections were attributed to the COVID-19 pandemic in the United States.^1^ Data from the Centers for Disease Control and Prevention revealed that members of racial/ethnic minority groups were disproportionately impacted by the COVID-19 pandemic, with Black, Hispanic, and American Indian or Alaska Native populations reporting increased rates of infection, hospitalization, and mortality compared with White populations. The COVID-19 pandemic has highlighted many of the long-standing disparities experienced by members of racial/ethnic minority groups. Racial/ethnic disparities found with COVID-19 are not associated solely with race/ethnicity and underlying health conditions, but also with social, structural, and environmental factors, such as food insecurity, as well as a long history of disparate treatment.^2,3^ The association between race/ethnicity and food insecurity is complex and intertwined with other factors that influence food insecurity, such as social and economic disadvantage, which are prevalent among racial/ethnic minority groups.^3^

The US Department of Agriculture (USDA) defines food insecurity as a “household-level economic and social condition of limited or uncertain access to adequate food.” This status includes lacking access to food, consuming foods high in calories and carbohydrates, eating food past the expiration date, and purchasing inexpensive and unhealthy foods. Food insecurity affects racial/ethnic minority groups disproportionately.^3^ From 2001 to 2016, the rates of food insecurity among Black, Hispanic, and American Indian or Alaska Native households were at least 2-fold those of White households, and the gap between these racial/ethnic minority groups and White populations persisted.^3^ According to the USDA Economic Research Service, the mean prevalence of food insecurity in the United States in 2019 was 10.5%. The rates of food insecurity among racial/ethnic minority groups were higher than the US mean rate, at 22.5% among Black populations and 18.5% among Hispanic populations.^4^

The objective of this study was to examine the associations of race/ethnicity and food insecurity with COVID-19 infection rates in the United States. The literature on the association between socioeconomic status (SES) and health disparities in the United States and recent analyses on factors associated with COVID-19 infection rates provide useful insights for understanding the associations we examined. A major finding of the SES literature was that underrepresented racial/ethnic groups (primarily Black, Hispanic, and American Indian or Alaska Native populations), low-income households, and individuals with underlying health conditions were the populations most at risk of contracting chronic or infectious diseases.^5,6,7^ Recent studies^8,9,10,11,12^ of COVID-19 infections and deaths found similar results. Thus, evidence suggests that several dimensions of social stratification, including race/ethnicity, income, education, occupation, and underlying conditions (or comorbidities), may be associated with health outcome disparities.

## Methods

This cross-sectional study used data sets that were aggregated at the county level, deidentified, and publicly available. The Pennsylvania State University Institutional Review Board (IRB) determined that this study did not require IRB approval and was exempt from informed consent owing to the use of publicly available data that were deidentified. This article is compliant with the Strengthening the Reporting of Observational Studies in Epidemiology (STROBE) reporting guideline for cross-sectional studies.

### Data Sources

We conducted a county-level, cross-sectional study that covered 3133 US counties. The spread of COVID-19 was measured by the cumulative positive infections for a 11-month period starting when the pandemic began in the US, from January 21 to December 14, 2020. This variable was obtained from publicly accessible New York Times coronavirus data, based on reports from state and local health agencies.^13^ Our 2 measures of food insecurity were limited access to healthy food (measured as a score out of 10, with 0 indicating highest access and 10 indicating lowest access) and the percentage of households in a county receiving Supplemental Nutrition Assistance Program (SNAP) benefits with low access to grocery stores. These measures were obtained from the 2020 County Health Rankings^14^ and the Food Environment Atlas,^15^ respectively. County-level data on racial/ethnic composition, as well as demographic data (ie, age and sex), socioeconomic characteristics (ie, median income and education), and geographic characteristics (ie, population density and rural population), were also obtained from the 2020 County Health Rankings based on self-reported data from the US Census Bureau.^14^ We calculated a health risk index as the first principal component of health risk factors (ie, obesity, diabetes, cardiovascular disease, and respiratory disease mortality) obtained from the Atlas of Rural and Small-Town America and the Institute for Health Metrics and Evaluation.^15,16,17^ Additional measures to capture nonhealth risk exposure included essential occupation status (ie, health care, sales, and transportation-related occupations) and overcrowded home status.^14,18^ Table 1 provides a detailed description of the variable definitions and data sources.

**Table 1.** Variable Definitions and Descriptions

Variable	Description	Source	Year
Race/ethnic composition			
Black	Percentage of county population	County Health Rankings 2020	2018
Hispanic	Percentage of county population	County Health Rankings 2020	2018
American Indian or Alaska Native	Percentage of county population	County Health Rankings 2020	2018
Asian American or Pacific Islander	Percentage of county population	County Health Rankings 2020	2018
White	Percentage of county population	County Health Rankings 2020	2018
Food insecurity			
Access to healthy food index score, 1-10	Limited access to healthy food, with 0 worst and 10 best	County Health Rankings 2020	2015
Household recipient of SNAP	Percentage of SNAP households with low access to grocery stores	Food Environment Atlas	2015
Demographic characteristics			
Persons aged ≥65 y	Percentage of county population	County Health Rankings 2020	2018
Women	Percentage of county population	County Health Rankings 2020	2018
Men	Percentage of county population	Athors’ calculation from County Health Rankings 2020	2018
Socioeconomic characteristic			
Median income	Median household income in US dollars	County Health Rankings 2020	2018
High school education and below	Percentage of county population with high school diploma or less	Atlas of Rural and Small-Town America	2014-2018
Risk exposure			
Health occupation	Percentage of county population who were essential workers in health care sector	American Community Survey	2014-2018
Sales occupation	Percentage of county population who were essential workers in sales sector	American Community Survey	2014-2018
Transportation occupation	Percentage of county population who were essential workers in transportation sector	American Community Survey	2014-2018
Overcrowded home	Percentage of households with overcrowded homes	County Health Rankings 2020	2012-2016
Health Risk Index	Percentage of county population with obesity	Atlas of Rural and Small-Town America	2013
	Percentage of county population with diabetes	Atlas of Rural and Small-Town America	2014
	Cardiovascular diseases mortality, per 100 000 residents	Institute for Health Metrics and Evaluation	2014
	Respiratory disease mortality, per 100 000 residents	Institute for Health Metrics and Evaluation	2014
Geographic characteristics			
Population density	Population density	Atlas of Rural and Small-Town America	2010
Rural population	Percentage of county population in rural areas	County Health Rankings 2020	2010
COVID-19 infection rate			
In July 2020	No. of COVID-19 infections per 1000 residents	Authors’ calculation from The New York Times	2020
In December 2020	No. of COVID-19 infections per 1000 residents	Authors’ calculation from The New York Times	2020

Abbreviation: SNAP, Supplemental Nutrition Assistance Program.

### Statistical Analysis

Using ordinary least squares, we estimated the associations of race/ethnicity and food insecurity with COVID-19 infection rates at 2 separate points: as of 6 months after the announcement of the first infections in the US (ie, July 21, 2020) and 11 months after the announcement (ie, December 14, 2020). Our covariates of interest included (1) the racial/ethnic composition of US counties by percentages of Black, Hispanic, American Indian or Alaska Native, and Asian American or Pacific Islander residents, (2) pre–COVID-19 measures of food insecurity (2 measures: limited access to healthy food and percentage of SNAP recipient households with limited access to grocery stores) to avoid the endogeneity of food insecurity due to the possible bidirectional association with COVID-19 infections,^19^ and (3) the interaction between race/ethnicity and food insecurity. To avoid collinearity, we set the proportion of the county population consisting of White residents as the reference group in the models. The coefficient estimates for racial/ethnic minority groups were therefore interpreted in comparison with the White majority population. Because race/ethnicity often serves as a proxy for economic and social conditions in the United States, disparities in health outcomes could be associated with differences in SES more broadly rather than with differences in race/ethnicity per se. To avoid this pitfall, we accounted for a broad set of potential confounders: demographic characteristics (ie, age and sex), economic factors (ie, household income and education attainment), health-related and nonhealth–related risk exposure (ie, comorbidities and essential occupations), and geographic characteristics (ie, population density and rural population). These covariates were standardized, which enabled us to interpret the coefficients in terms of the contribution of each predictor to the number of infections per 1000 residents associated with a 1-SD change in the predictor of interest.

The ordinary least squares estimation we used to identify the association of race/ethnicity and food insecurity with COVID-19 infections in the US was as follows:

*C_is_* = β_0_ + β_1_*R_is_* + β_2_*F_is_* + β_3_*R_is_ × F_is_* + β_4_*X_is_* + δ*_s_* + ε*_is_*

where *C* indicates the number of COVID-19 infections per 1000 residents (hereafter *infections*) in a county; *R* represents the racial/ethnic composition of the county-level population; *F* is a measure of county-level food insecurity; *X* is a vector of demographic, socioeconomic, and geographic county-level characteristics; δ represents the state fixed effects; and *i* and *s* are the county and state indicators, respectively. In all model specifications, SEs were robust to heteroskedasticity and clustered at the state level to control for correlations among counties in each state. Given the large number of covariates, multicollinearity diagnostic tests using variance inflation factors were conducted. Supplemental tables (eTable 1 and eTable 2 in the Supplement) were included to demonstrate the process of variable selection based on the Akaike information criterion and Bayesian information criterion. Stata statistical software version 16 (StataCorp) was used for data analysis. *P* values were 2-sided, and statistical significance was set at *P* < .05. Data were analyzed from November 2020 through March 2021.

## Results

A total of 3133 US counties were included in the analysis. The sample size was reduced because of missing values for limited access to healthy food and COVID-19 infections for July 2020. We had 3096 observations for COVID-19 infections in July 2020 (ie, 37 missing values) and 3114 observations for the variable limited access to healthy food (ie, 19 missing values); all other variables had 3133 observations. The estimated sample sizes for the models varied by the number of missing values and their combination in specific models: model 1: 3077 observations; model 2: 3114 observations; model 3: 3096 observations; and model 4: 3133 observations. Table 2 presents summary statistics of the socioeconomic and demographic characteristics of the counties together with their risk exposure and geographic characteristics. The mean (SD) racial/ethnic composition indicated that 9.0% (14.3%) of the population were Black residents, 9.6% (13.8%) were Hispanic residents, 2.3% (7.3%) were American Indian or Alaska Native residents, 1.7% (3.2%) were Asian American or Pacific Islander residents, and 76.1% (20.1%) were White residents. The mean (SD) proportion of women was 49.9% (2.3%), and the mean (SD) proportion of individuals aged 65 years or older was 19.3% (4.7%). The mean (SD) infections were 8.1 (9.2) in July 2020 and 56.7 (26) in December 2020. The mean (SD) score for limited access to healthy food was 2.5 (1.1), while the mean (SD) percentage of SNAP recipient households with low access to grocery stores was 2.9% (3.0%).

**Table 2.** Summary Statistics

Characteristic	Mean (SD) [Range]
Racial/ethnic composition	
Black, %	9 (14.3) [0-85.4]
Hispanic, %	9.6 (13.8) [0.6-96.4]
American Indian or Alaska Native, %	2.3 (7.3) [0-85.7]
Asian American or Pacific Islander, %	1.7 (3.2) [0-52.6]
White, %	76.1 (20.1) [2.7-97.9]
Food insecurity	
Access to healthy food	7.5 (1.1) [0-10.0]
Household recipient of SNAP, %	2.9 (3) [0-37.3]
Demographic	
Persons aged ≥65 y, %	19.3 (4.7) [4.8-57.6]
Women, %	49.9 (2.3) [26.8-56.9]
Men, %	50.1 (2.3) [43.1-73.2]
Socioeconomic	
Median income, $	52 800.0 (13 878.6) [25 385.0-140 382.0]
High school education and below, %	47.7 (10.7) [6.7-87.4]
Risk exposure	
Health occupation, %	1.5 (0.7) [0-6.7]
Sales occupation, %	4.1 (1.1) [0-9.0]
Transportation occupation, %	2 (0.7) [0-7.9]
Overcrowded home, %	2.4 (2.2) [0-38.0]
Geographic	
Population density	259.9 (1726.8) [0-69 468.4]
Rural population, %	58.6 (31.5) [0-100]
COVID-19 infection rate, per 1000 residents	
July 2020	8.1 (9.2) [0-140.5]
December 2020	56.7 (26.0) [0-244.1]

Abbreviation: SNAP, Supplemental Nutrition Assistance Program.

Our regression results are presented in Table 3. For each measure of food insecurity, we estimated the factors associated with COVID-19 infections as of July 21 and December 14, 2020. For July 21, 2020, with limited access to healthy food as our measure of food insecurity, we found that a 1-SD increase in the percentage of Black and Hispanic residents in a county was associated with an increase in infections of 2.99 (95% CI, 2.04 to 3.94; *P* < .001) and 2.91 (95% CI, 0.39 to 5.43; *P* = .02), respectively, even after controlling for other confounders of COVID-19 infection. By contrast, a 1-SD increase in the percentage of American Indian or Alaska Native residents and Asian American or Pacific Islander residents in a county was not associated with infections, with changes in infections of 0.16 (95% CI; −0.73 to 1.04; *P* = .73) and 0.44 (95% CI, −0.50 to 1.37; *P* = .35) infections, respectively. We found that there was a decrease in COVID-19 infections (coefficient, −0.36; 95% CI, −0.87 to 0.15; *P* = .17) with decreased access to healthy food overall as of July 21, 2020, but this difference was not statistically significant.

**Table 3.** Factors Associated With COVID-19 Infection

Factor^a^	Limited access to healthy food				SNAP recipient household with limited access to food			
	July 2020		December 2020		July 2020		December 2020	
	Coefficient (95% CI)	*P* value	Coefficient (95% CI)	*P* value	Coefficient (95% CI)	*P* value	Coefficient (95% CI)	*P* value
Racial/ethnic composition								
Black, %	2.99 (2.04 to 3.94)	<.001	0.66 (−2.02 to 3.34)	.62	3.24 (2.46 to 4.02)	<.001	0.78 (−1.20 to 2.76)	.43
Hispanic, %	2.91 (0.39 to 5.43)	.02	5.64 (3.54 to 7.75)	<.001	3.62 (0.90 to 6.35)	.01	7.18 (4.94 to 9.43)	<.001
American Indian or Alaska Native, %	0.16 (−0.73 to 1.04)	.73	1.52 (−0.29 to 3.33)	.10	0.63 (−0.63 to 1.88)	.32	2.26 (0.44 to 4.09)	.02
Asian American or Pacific Islander, %	0.44 (−0.50 to 1.37)	.35	−1.39 (−2.29 to −0.49)	.003	0.44 (−0.53 to 1.40)	.37	−1.33 (−2.17 to −0.48)	.003
Food insecurity by race/ethnicity								
Food insecurity	−0.36 (−0.87 to 0.15)	.17	−2.66 (−4.52 to −0.81)	.006	−0.54 (−1.03 to −0.05)	.03	−2.16 (−3.54 to −0.79)	.003
Black population × food insecurity	0.16 (−0.28 to 0.59)	.48	0.90 (0.33 to 1.47)	.003	0.06 (−0.29 to 0.41)	.75	0.87 (0.46 to 1.29)	<.001
Hispanic population × food insecurity	−0.69 (−1.25 to −0.12)	.02	−0.50 (−1.28 to 0.29)	.21	−0.53 (−0.97 to −0.09)	.02	−0.54 (−0.92 to −0.16)	.006
American Indian or Alaska Native population × food insecurity	0.20 (−0.17 to 0.57)	.29	0.57 (0.06 to 1.08)	.03	0.02 (−0.25 to 0.28)	.89	0.25 (−0.05 to 0.55)	.11
Asian American or Pacific Islander population × food insecurity	−0.40 (−0.91 to 0.11)	.12	−1.48 (−2.26 to −0.70)	<.001	−0.43 (−1.05 to 0.19)	.17	−1.51 (−2.20 to −0.83)	<.001
Demographic characteristics								
Persons aged ≥65 y, %	−0.57 (−1.09 to −0.05)	.03	−3.13 (−4.20 to −2.06)	<.001	−0.51 (−1.04 to 0.02)	.06	−2.74 (−3.92 to −1.57)	<.001
Women, %	−0.75 (−1.67 to 0.16)	.11	−4.51 (−6.84 to −2.18)	<.001	−0.94 (−2.18 to 0.30)	.14	−4.64 (−7.08 to −2.20)	<.001
Socioeconomic characteristics								
Median income, $	0.33 (−0.46 to 1.13)	.41	−3.35 (−4.83 to −1.87)	<.001	0.46 (−0.26 to 1.18)	.21	−2.18 (−3.84 to −0.52)	.01
High school education and below, %	1.90 (0.74 to 3.06)	.002	3.47 (1.20 to 5.74)	.003	1.78 (0.44 to 3.13)	.01	3.79 (1.44 to 6.14)	.002
Health and nonhealth risk factors								
Health risk index	−0.53 (−1.51 to 0.45)	.28	2.34 (0.42 to 4.26)	.02	−0.34 (−1.59 to 0.91)	.59	2.58 (0.50 to 4.65)	.02
Health occupation, %	0.33 (−0.06 to 0.72)	.10	2.12 (1.09 to 3.15)	<.001	0.25 (−0.17 to 0.66)	.24	2.15 (1.14 to 3.16)	<.001
Sales occupation, %	0.48 (0.23 to 0.72)	<.001	0.27 (−0.55 to 1.10)	.51	0.52 (0.20 to 0.84)	.002	0.31 (−0.54 to 1.15)	.47
Transportation occupation, %	−0.51 (−0.98 to −0.04)	.03	−0.09 (−1.28 to 1.10)	.88	−0.62 (−1.09 to −0.15)	.01	−0.22 (−1.39 to 0.94)	.70
Overcrowded home, %	0.83 (−0.77 to 2.43)	.30	−0.13 (−1.30 to 1.03)	.82	0.92 (−0.64 to 2.48)	.24	−0.01 (−1.28 to 1.26)	.99
Geographic characteristics								
Population density	0.13 (−0.14 to 0.40)	.33	0.18 (−0.06 to 0.42)	.13	0.04 (−0.24 to 0.32)	.79	−0.09 (−0.35 to 0.17)	.49
Rural population, %	−0.41 (−0.88 to 0.06)	.09	−3.75 (−5.04 to −2.46)	<.001	−0.30 (−0.87 to 0.27)	.30	−3.73 (−5.09 to −2.38)	<.001
Constant term^b^	14.60 (12.87 to 16.33)	<.001	59.70 (56.50 to 62.90)	<.001	14.98 (13.09 to 16.87)	<.001	60.83 (57.57 to 64.09)	<.001
Observations, No.^c^	3077	NA	3114	NA	3096	NA	3133	NA
Adjusted *R^2^*	0.453	NA	0.594	NA	0.430	NA	0.594	NA
Variance inflation factor, mean	2.46	NA	2.46	NA	2.36	NA	2.36	NA
Highest variance inflation factor	5.21	NA	5.22	NA	5.75	NA	5.66	NA
State fixed effect	Yes	NA	Yes	NA	Yes	NA	Yes	NA

Abbreviation: NA, not applicable; SNAP, Supplemental Nutrition Assistance Program.

^a^ Point estimates are expressed in number of infections per 1000 residents.

^b^ The coefficient is the expected COVID-19 infection rate per 1000 residents if all independent variables were equal to 0.

^c^ Observations are the number of counties in each estimation.

Furthermore, we examined the extent to which the association between racial/ethnic composition and COVID-19 infections interacted with food insecurity. Our finding from this interaction suggested that counties with poor access to healthy food and large Black populations and American Indian or Alaska Native populations had increased infection. However, the interaction suggested that in counties with poor access to healthy food, for every 1-SD increase in the percentage of Black residents or the percentage of American Indian or Alaska Native residents, there was an increase in infections of 0.16 (95% CI, −0.28 to 0. 59; *P* = .48) and 0.20 (95% CI, −0.17 to 0.57; *P* = .29), respectively. These increases, however, were not statistically significant. Conversely, in counties with poor access to healthy food, a 1-SD increase in the percentage of Hispanic residents was associated with decreased infection of −0.69 (95% CI, −1.25 to −0.12; *P* = .02). We found similar results using the percentage of SNAP recipient households with low access to grocery stores as our alternative measure of food insecurity.

As of December 2020, the results were markedly different. The coefficient for the proportion of Black residents in a county had decreased by 4.53-fold, to 0.66 (95% CI, −2.02 to 3.34; *P* = .62) infections, and was no longer statistically significant (Table 3). In other words, an increased percentage of Black residents in a county was no longer associated with increased COVID-19 infections as it had been in July 2020. However, we found a larger increase in COVID-19 infection associated with Hispanic and American Indian or Alaska Native populations compared with July 2020. Specifically, we found that a 1-SD increase in the percentage of Hispanic residents in a county was associated with a 1.94-fold increase in infection (coefficient, 5.64; 95% CI, 3.54 to 7.75; *P* < .001) compared with July 2020. Meanwhile, for every 1-SD increase in the percentage of American Indian or Alaska Native residents in a county, there was a 9.50-fold increase in infection compared with July 2020, but the coefficient (1.52; 95% CI, −0.29 to 3.33; *P* = .10) was not statistically significant. On the other hand, a 1-SD increase in the percentage of Asian American or Pacific Islander residents in a county was associated with a decrease in infections of −1.39 (95% CI, −2.29 to 0.49; *P* = .003). This contrasts with the increase in infections with every 1-SD increase in the percentage of this population in a county found in July 2020, which was not statistically significant (Table 3).

Furthermore, the association of COVID-19 infections with food insecurity by race/ethnicity had also noticeably changed by December 2020. Results showed that a 1-SD decrease in access to healthy food (or equivalently here, a 1-SD increase in food insecurity) was associated with an increase of 0.90 (95% CI; 0.33 to 1.47; *P* = .003) infections in counties with large percentages of Black residents and 0.57 (95% CI, 0.06 to 1.08; *P* = .03) infections in counties with large percentages of American Indian or Alaska Native residents. In contrast, a 1-SD decrease in access to healthy food was associated with fewer infections in counties with large proportions of Asian American or Pacific Islander residents (interaction coefficient, −1.48; 95% CI, −2.26 to −0.70; *P* < .001). However, it was not associated with differences in infections in counties with increased percentages of Hispanic residents (interaction coefficient, −0.50; 95% CI, −1.28 to 0.29; *P* = .21).

Overall, the confounding factors showed expected results. Counties with a large proportion of residents with low education attainment and increased health and occupational risk (ie, working in the health care sector and sales) had increased infection rates compared with counties with lower levels of these characteristics. Conversely, counties with a large proportion of women, residents aged 65 years and older, and households with high income had decreased infection rates. Similar results were obtained using the percentage of SNAP recipient households with low access to grocery stores as our measure of food insecurity (Table 3).

## Discussion

This cross-sectional study found that the associations of race/ethnicity and food insecurity with COVID-19 infections varied among racial/ethnic minority groups and over time in the 11 months after the first COVID-19 infections were reported in the United States. There were markedly different outcomes in December vs July 2020. For the initial 6 months of the pandemic, we found an association between the proportion of residents from several minority groups (ie, the proportion of Black and Hispanic residents) in a county’s population and COVID-19 infections but no systematic association of food insecurity with infections, either independently or in interaction with most minority groups. Conversely, we found that by December 2020 the percentage of Black residents in a county was no longer associated with infections. However, in counties with large Black populations or large American Indian or Alaska Native populations, the interaction with food insecurity was positive and significant, indicating an increase in infections. This is in line with evidence suggesting that poverty was an aggravating factor associated with increased rates of ICU admission among Black patients with COVID-19.^20^

However, the increase in infections was greater (5.64 more infections per 1000 residents in December vs 2.91 in July) and remained statistically significant (95% CI, 3.54 to 7.75; *P* < .001) in counties with a large proportion of Hispanic residents. This could be partly associated with the particularly rapid rise in COVID-19 infections among Hispanic communities in the second half of 2020, as the disease spread relentlessly in states with large Hispanic populations, such as California, Texas, Florida, and Arizona. Overall, these findings are consistent with evidence of the intersectionality among race/ethnicity, socioeconomic determinants of health, and the persistent associations of food insecurity among people of color^3^ and study findings that the association of food insecurity with health outcomes differs by racial/ethnic group.^21,22,23,24^

Our study highlights county-level associations between race/ethnicity and risk of COVID-19, along with the interaction with food insecurity. We found an increased prevalence of COVID-19 in counties with large racial/ethnic minority populations compared with the majority White populations, which is consistent with existing literature examining racial/ethnic disparities in COVID-19 infections. We acknowledge that the disparities found in terms of race/ethnicity may be attributed to social, environmental, and structural factors that are associated with increased risk of COVID-19 infection in minority groups; these factors include decreased access to testing, residing in multifamily and multigenerational households, reliance on public transportation, and increased rates of chronic disease.

The prevalence of food insecurity has increased during the pandemic. There is an emerging literature that examines the association of COVID-19 with prevailing food insecurity levels across racial/ethnic groups. A study by Webb Hooper et al^22^ found that Black and Hispanic populations bore a disproportionately higher burden of COVID-19 outcomes in the initial months of the COVID-19 pandemic. Other studies found that Black populations experienced increased rates of positive diagnosis and loss of employment compared with White populations,^2^ food insecurity increased across all racial/ethnic groups but minority groups were more likely to experience difficulty in buying food,^23^ and COVID-19 was associated with increases in existing health disparities related to food security.^24^ Of consequence, however, is that these studies highlight the challenges of food insecurity by race/ethnicity and the need for increased food assistance programs and support services to improve the food supply chain.

### Limitations

This study has several limitations. It did not explore the need for food assistance programs or support services, because we used pre–COVID-19 measures of food insecurity. In addition to the paucity of relevant county-level food insecurity data across US counties during the pandemic, there is a need to address the endogeneity associated with policy measures aimed at stemming the spread of COVID-19, such as lockdown, and diagnosis of and death due to COVID, which are significant drivers of the prevailing food insecurity found among these populations. In this study, we avoided this limitation by using food insecurity data from a period before the COVID-19 pandemic.

## Conclusions

The findings of this cross-sectional study of the association of race/ethnicity with COVID-19 infection rates and the interaction of pre-COVID experiences of food insecurity suggest that the association varied over time and across racial/ethnic groups. While in July, race/ethnicity seemed to be the primary factor associated with increased COVID-19 infections, especially among Black and Hispanic populations, by mid-December, there was no association between large Black populations and COVID-19 infections, but there was an interaction between race/ethnicity and food insecurity. While counties with a large percentage of Hispanic residents had increased COVID-19 infections, counties with large Black or large American Indian or Alaska Native populations had increased COVID-19 infections only when they had increased levels of food insecurity. Counties with large proportions of Asian residents, on the other hand, had decreased COVID infection rates even when they had increased rates of food insecurity. Although our results highlight a unique association between race/ethnicity and COVID-19 infection at various points of the pandemic, our county-level study is currently unable to address the documented association of food insecurity with COVID-19 infection during the pandemic, partly owing to a lack of county-level data on food insecurity during the COVID-19 pandemic and owing to our study’s research design. Further research is needed to examine the bidirectional association between food insecurity during the pandemic and COVID-19 infection.

## Notes

Supplement. **eTable 1.** Model Selection With Limited Access to Healthy Food: December 2020 **eTable 2.** Model Selection With SNAP Recipients: December 2020
