# Supplementary material for: Associations of Race/Ethnicity and Food Insecurity With COVID-19 Infection Rates Across US Counties
Source: JAMA Netw Open. 2021 Jun 8;4(6):e2112852. doi: 10.1001/jamanetworkopen.2021.12852 (PMC8188266; doi:10.1001/jamanetworkopen.2021.12852)
Supplement: Supplement. — eTable 1. Model Selection With Limited Access to Healthy Food: December 2020 eTable 2. Model Selection With SNAP Recipients: December 2020 [file jamanetwopen-e2112852-s001.pdf]

# Supplemental Online Content

Kimani ME, Sarr M, Cuffee Y, Liu C, Webster NS. Associations of race/ethnicity and food insecurity with COVID-19 infection rates across US counties. *JAMA Netw Open*. 2021;4(6):e2112852. doi:10.1001/jamanetworkopen.2021.12852

**eTable 1.** Model Selection With Limited Access to Healthy Food: December 2020

**eTable 2.** Model Selection With SNAP Recipients: December 2020

This supplemental material has been provided by the authors to give readers additional information about their work.

© 2020 Kimani ME et al. *JAMA Network Open*.

**eTable 1.** Model Selection With Limited Access to Healthy Food: December 2020

| | Race/Ethnicity Only | | Add Food Insecurity | | Add Interactions | | Add Confounders | |
|---|---|---|---|---|---|---|---|---|
| Factor [a] | Coefficient (95% CI) | P-value | Coefficient (95% CI) | P-value | Coefficient (95% CI) | P-value | Coefficient (95% CI) | P-value |
| *Racial/Ethnic composition* | | | | | | | | |
| Black, % | 3.52 (1.71 to 5.33) | <.001 | 1.88 (-1.69 to 5.45) | .30 | 3.06 (0.43 to 5.69) | .02 | 0.66 (-2.02 to 3.34) | .62 |
| Hispanic, % | 9.60 (7.05 to 12.15) | <.001 | 3.21 (1.10 to 5.33) | .004 | 9.36 (7.13 to 11.59) | <.001 | 5.64 (3.54 to 7.75) | <.001 |
| American Indian or Alaska Native, % | 3.53 (2.37 to 4.70) | <.001 | 5.07 (1.17 to 8.96) | .01 | 2.90 (1.41 to 4.39) | <.001 | 1.52(-0.29 to 3.33) | .10 |
| Asian American or Pacific Islander, % | -0.89 (-1.74 to -0.04) | .04 | -4.59 (-5.83 to -3.35) | <.001 | -0.94 (-1.78 to -0.10) | .03 | -1.39(-2.29 to -0.49) | .003 |
| *Food Insecurity by race/ethnicity* | | | | | | | | |
| Food Insecurity | | | -2.05 (-6.30 to 2.21) | .34 | -1.19 (-3.48 to 1.10) | .30 | -2.66(-4.52 to -0.81) | .006 |
| Black population × food insecurity | | | | | 0.60 (-0.02 to 1.22) | .06 | 0.90(0.33 to 1.47) | .003 |
| Hispanic population × food insecurity | | | | | -1.04 (-1.87 to -0.22) | .01 | -0.50(-1.28 to 0.29) | .21 |
| American Indian or Alaska Native × food insecurity | | | | | 0.36 (-0.04 to 0.76) | .07 | 0.57 (0.06 to 1.08) | .03 |
| Asian American or Pacific Islander × food insecurity | | | | | 0.04 (-0.99 to 1.08) | .93 | -1.48 (-2.26 to -0.70) | <.001 |
| *Demographics* | | | | | | | | |
| Persons aged ≥ 65y, % | | | | | | | -3.13 (-4.20 to -2.06) | <.001 |
| Women, % | | | | | | | -4.51 (-6.84 to -2.18) | <.001 |
| *Socio-economic characteristics* | | | | | | | | |
| Median income, dollars | | | | | | | -3.35 (-4.83 to -1.87) | <.001 |
| High school education and below, % | | | | | | | 3.47 (1.20 to 5.74) | .003 |
| *Health & Non-health risk factors* | | | | | | | | |
| Health risk index | | | | | | | 2.34 (0.42 to 4.26) | .02 |
| Health occupations, % | | | | | | | 2.12 (1.09 to 3.15) | <.001 |
| Sales occupations, % | | | | | | | 0.27 (-0.55 to 1.10) | .51 |
| Transportation occupations, % | | | | | | | -0.09 (-1.28 to 1.10) | .88 |
| Overcrowded homes, % | | | | | | | -0.13 (-1.30 to 1.03) | .82 |
| *Geographic characteristics* | | | | | | | | |
| Population density | | | | | | | 0.18 (-0.06 to 0.42) | .13 |
| Rural, % | | | | | | | -3.75 (-5.04 to -2.46) | <.001 |
| Constant term [b] | 62.5 (59.68 to 65.33) | <.001 | 56.68 (51.61 to 61.74) | <.001 | 61.81 (58.68 to 64.94) | <.001 | 59.7 (56.50 to 62.90) | <.001 |
| Observations, No. [c] | 3142 | NA | 3123 | NA | 3123 | NA | 3114 | NA |
| Adjusted R-square | 0.524 | NA | 0.065 | NA | 0.529 | NA | 0.594 | NA |
| AIC | 27024.33 | NA | 29003.43 | NA | 26815.37 | NA | 26267.04 | NA |
| BIC | 27048.54 | NA | 29039.71 | NA | 26869.79 | NA | 26387.92 | NA |

© 2020 Kimani ME et al. *JAMA Network Open*.

| | | | | | | | | |
|---|---|---|---|---|---|---|---|---|
| State Fixed Effect | Yes | NA | Yes | NA | Yes | NA | Yes | NA |

Abbreviation: NA, not applicable; SNAP, Supplemental Nutritional Assistance Program; AIC, Akaike Information Criterion; BIC, Bayesian Information Criterion.

[a] Point estimates are expressed in number of infections per 1000 residents.

[b] The coefficient is the expected COVID-19 infection rate per 1000 residents if all independent variables were equal to 0.

[c] Observations are the number of counties in each estimation.

© 2020 Kimani ME et al. *JAMA Network Open*.

**eTable 2.** Model Selection With SNAP Recipients: December 2020

| | Race/Ethnicity Only | | Add Food Insecurity | | Add Interactions | | Add Confounders | |
|---|---|---|---|---|---|---|---|---|
| Factor [a] | Coefficient (95% CI) | P-value | Coefficient (95% CI) | P-value | Coefficient (95% CI) | P-value | Coefficient (95% CI) | P-value |
| *Racial/Ethnic composition* | | | | | | | | |
| Black, % | 3.52 (1.71 to 5.33) | <.001 | 1.15 (-2.02 to 4.31) | .47 | 3.38 (1.61 to 5.15) | <.001 | 0.78 (-1.20 to 2.76) | .43 |
| Hispanic, % | 9.60 (7.05 to 12.15) | <.001 | 3.23 (0.87 to 5.58) | .008 | 10.70 (8.44 to 12.96) | <.001 | 7.18 (4.94 to 9.43) | <.001 |
| American Indian or Alaska Native, % | 3.53 (2.37 to 4.70) | <.001 | 4.81 (1.34 to 8.28) | .008 | 3.41 (1.76 to 5.07) | <.001 | 2.26 (0.44 to 4.09) | .02 |
| Asian American or Pacific Islander, % | -0.89 (-1.74 to -0.04) | .04 | -4.44 (-5.60 to -3.28) | <.001 | -1.31 (-2.20 to -0.41) | .005 | -1.33 (-2.17 to -0.48) | 0.003 |
| *Food Insecurity by race/ethnicity* | | | | | | | | |
| Food Insecurity | | | -1.02 (-3.24 to 1.21) | .36 | -1.70 (-3.29 to -0.12) | .04 | -2.16 (-3.54 to -0.79) | .003 |
| Black population × food insecurity | | | | | 0.66 (0.07 to 1.26) | .03 | 0.87 (0.46 to 1.29) | <.001 |
| Hispanic population × food insecurity | | | | | -0.80 (-1.20 to -0.41) | <.001 | -0.54 (-0.92 to -0.16) | .006 |
| American Indian or Alaska Native × food insecurity | | | | | 0.24 (-0.05 to 0.53) | .10 | 0.25 (-0.05 to 0.55) | .11 |
| Asian American or Pacific Islander × food insecurity | | | | | -0.38 (-0.92 to 0.16) | .17 | -1.51 (-2.20 to -0.83) | <.001 |
| *Demographics* | | | | | | | | |
| Persons aged ≥ 65y, % | | | | | | | -2.74 (-3.92 to -1.57) | <.001 |
| Women, % | | | | | | | -4.64 (-7.08 to -2.20) | <.001 |
| *Socio-economic characteristics* | | | | | | | | |
| Median income, dollars | | | | | | | -2.18 (-3.84 to -0.52) | .01 |
| High school education and below, % | | | | | | | 3.79 (1.44 to 6.14) | .002 |
| *Health & Non-health risk factors* | | | | | | | | |
| Health risk index | | | | | | | 2.58 (0.50 to 4.65) | .02 |
| Health occupations, % | | | | | | | 2.15 (1.14 to 3.16) | <.001 |
| Sales occupations, % | | | | | | | 0.31 (-0.54 to 1.15) | .47 |
| Transportation occupations, % | | | | | | | -0.22 (-1.39 to 0.94) | .70 |
| Overcrowded homes, % | | | | | | | -0.01 (-1.28 to 1.26) | .99 |
| *Geographic characteristics* | | | | | | | | |
| Population density | | | | | | | -0.09 (-0.35 to 0.17) | .49 |
| Rural, % | | | | | | | -3.73 (-5.09 to -2.38) | <.001 |
| Constant term [b] | 62.5 (59.68 to 65.33) | <.001 | 56.68 (51.56 to 61.79) | <.001 | 62.32 (59.39 to 65.25) | <.001 | 60.83 (57.57 to 64.09) | <.001 |
| Observations, No. [c] | 3142 | NA | 3140 | NA | 3140 | NA | 3133 | NA |

© 2020 Kimani ME et al. *JAMA Network Open*.

| | | | | | | | | |
|---|---|---|---|---|---|---|---|---|
| Adjusted $R^2$ | 0.524 | NA | 0.060 | NA | 0.529 | NA | 0.594 | NA |
| AIC | 27024.33 | NA | 29188.99 | NA | 26969.08 | NA | 26445.77 | NA |
| BIC | 27048.54 | NA | 29225.30 | NA | 27023.55 | NA | 26566.77 | NA |
| State Fixed Effect | Yes | NA | Yes | NA | Yes | NA | Yes | NA |

Abbreviation: NA, not applicable; SNAP, Supplemental Nutritional Assistance Program; AIC, Akaike Information Criterion; BIC, Bayesian Information Criterion.
[a] Point estimates are expressed in number of infections per 1000 residents.
[b] The coefficient is the expected COVID-19 infection rate per 1000 residents if all independent variables were equal to 0.
[c] Observations are the number of counties in each estimation.

© 2020 Kimani ME et al. *JAMA Network Open*.
